# Prognostic scores for predicting overall survival in patients with metastatic renal and urothelial cancer undergoing immunotherapy - which one to use?

**DOI:** 10.1007/s00345-025-05452-4

**Published:** 2025-01-29

**Authors:** Margarete Teresa Walach, Ralph Burger, Felix Brumm, Katja Nitschke, Frederik Wessels, Philipp Nuhn, Thomas Stephan Worst, Jost von Hardenberg, Britta Grüne, Jonas Jarczyk

**Affiliations:** 1 Department of Urology and Urologic Surgery, University Medical Centre Mannheim (UMM), University of Heidelberg, Theodor-Kutzer-Ufer 1-3, 68167 Mannheim, Germany; 2 Department of Urology, University of Kiel (UKSH), Arnold-Heller-Strasse 1-3, 24105 Kiel, Germany

**Keywords:** Metastatic urothelial carcinoma, Metastatic renal cell carcinoma, Immunotherapy, Prognostic score, mGPS, NLR, SII, NER

## Abstract

**Purpose:**

Evaluation of the prognostic significance of four different scoring systems in a real-world cohort of patients with metastatic urothelial carcinoma (mUC) or renal cell carcinoma (mRCC) undergoing immunotherapy (IO).

**Methods:**

For 120 patients with mUC (*n* = 67) and mRCC (*n* = 53) who received IO between July 2016 and December 2020 at the tertiary Urological University Medical Centre Mannheim, the following scores were recorded at pre-treatment baseline: modified Glasgow prognostic score (mGPS), systemic immune-inflammation index (SII), neutrophil-to-lymphocyte ratio (NLR), neutrophil-to-eosinophil ratio (NER). Overall survival (time between the beginning of IO until the patients’ death or last contact) was determined for every patient.

**Results:**

Kaplan-Meier analyses revealed that high baseline mGPS, SII (> 979) and NLR (> 3) were associated with poor overall survival (OS) (*p* < 0.05). Cox proportional hazards regression analyses showed that baseline mGPS and NLR had a significant independent prognostic influence on OS (*p* < 0.05), of which mGPS had a greater significance (*p* < 0.001, mGPS Score 2 vs. Score 0: HR 4.1, 95% CI 1.9–8.8). Although a high baseline NER (63.9) was associated with poor OS, it did not reach statistical significance. Baseline NER was also not identified as a significant score in the regression analyses.

**Conclusion:**

mGPS, SII and NLR are scoring systems that are easy to record in routine clinical practice. As they provide good prediction of OS in patients with mUC and mRCC under IO, they may allow identification of patients at high-risk and monitor them more cautiously in addition to imaging.

**Supplementary Information:**

The online version contains supplementary material available at 10.1007/s00345-025-05452-4.

## Introduction

Immunotherapy (IO) has become standard of care for the treatment of urological malignancies such as metastatic renal cell (mRCC) and urothelial carcinoma (mUC). This also applies to urothelial carcinoma following the approval of the combination therapy of enfortumab vedotin and pembrolizumab as first-line therapy [1, 2]. IO is associated with manageable adverse events and is generally well tolerated [3]. However, IO has increased the healthcare burden enormously and not every patient will benefit from it [4]. Therefore, in addition to molecular biomarkers such as PD-L1, many scoring systems using blood biomarkers have been investigated more closely in a clinical setting, with the scope to predict response to IO in urological malignancies [5–8].

In a phase IIIB trial (*SAUL*) investigating the safety of atezolizumab in the treatment of mUC, the neutrophil-to-lymphocyte ratio (NLR) and systemic immune-inflammation Index (SII) were prognostic for progression free survival (PFS) and overall survival (OS), with high NLR and high SII being associated with worse PFS and OS [5]. Consistently, a large multi-center study (*ARON-1*) including 1265 mRCC patients treated with first-line immune combination showed that low SII was associated with longer OS and better PFS, as well as higher overall response rate (ORR) [6]. In mRCC, studies investigating the IO/IO combination ipilimumab/nivolumab demonstrated that lower neutrophil-to-eosinophil ratio (NER) was associated with improved OS, PFS and ORR [9]. These results underline the idea that high eosinophil count is associated with reduced risk of disease progression in mRCC treated with nivolumab [10]. Furthermore, modified Glasgow prognostic score (mGPS) has been highlighted as interesting candidate tool evaluating its prognostic value in urological cancers [7, 11]. It could be demonstrated that the integration of mGPS in therapy monitoring of mRCC patients treated with atezolizumab/bevacizumab or sunitinib might provide valuable prognostic information additionally to radiologic staging [8].

There is currently no universally accepted risk score in metastatic urological cancers, particularly in the era of IO. To our knowledge, NLR, SII, NER and mGPS are the most studied scoring systems attempting to predict IO treatment response. Therefore, we focused on evaluating the proposed scoring systems in a real-world cohort of mRCC and mUC patients treated with IO and assessing which of the mentioned scores is most reliable to be used in routine clinical practice. With the support of well-defined, easy-to-use and evidence-based scoring systems, costly and non-responsive overtreatment could be avoided and early therapy optimization might be feasible.

## Material & methods

### Study design, study population and data collection

Data of 122 patients with mRCC or mUC receiving IO at the tertiary Urological University Medical Centre Mannheim between July 2016 and December 2020 were retrospectively assessed. Next to clinical and oncologic variables, laboratory parameters, such as leukocytes, thrombocytes, neutrophils, eosinophils, lymphocytes, albumin and CRP were recorded.

In May 2022 the patients’ survival status was obtained from the local tumor register. Consequently, this date marks the endpoint of survival analysis. The study was conducted according to the Declaration of Helsinki. Ethical approval was obtained from the University of Heidelberg’s Ethics Committee II (Medical Faculty Mannheim, reference number 2015–549 N-MA).

### Scores and outcomes

Four combined scores were evaluated as prognostic biomarkers: mGPS, SII, NLR and NER. The scores were calculated as follows: mGPS: C-reactive protein < 10 mg/l = 0; C-reactive protein > 10 mg/l and albumin > 35 g/l = 1; C-reactive protein > 10 mg/l and albumin < 35 g/l = 2. SII: (neutrophil count x platelet count)/lymphocyte count. NLR: neutrophil count/lymphocyte count. NER: neutrophil count/eosinophil count. For baseline mGPS, SII, NLR and NER, laboratory parameters of the day of the first treatment or of the visit before were used (median (IQR), 0 (0–2) days). The primary outcome of this study was OS. It was defined as the time between beginning of IO until patients’ death or last contact.

### Statistical analyses

Descriptive statistics were performed to present baseline demographics and clinical characteristics of patients. Continuous variables are expressed as median and interquartile range (IQR), and categorical variables as absolute and relative frequencies. Baseline characteristics were compared between patients with mUC and mRCC using Pearson’s χ² test, Fisher’s exact test or Mann-Whitney test. Receiver operating curve (ROC) analyses for SII, NLR and NER were used to calculate the area under the curve (AUC) and determine optimal cut-off values, their sensitivity and specificity (using the Youden’s index). OS probability of baseline mGPS, SII, NLR and NER was presented in Kaplan-Meier curves and compared with log-rank test.

Uni- and multivariate Cox proportional hazards regression analyses were performed to compare the prognostic value of the four scores with regard to OS. Only independent variables with a p value < 0.05 on univariate logistic regression were included in the multivariate model. All statistical analyses were carried out with JMP^®^ v16 (SAS Institute, Cary, NC, USA). The level of significance was set at 0.05.

## Results

### Patient characteristics

A total of 122 patients receiving IO at the study centre were identified. After excluding two patients with pure squamous cell carcinoma of the urinary bladder, 120 patients were included, 67 (55.8%) with mUC and 53 (44.2%) with mRCC.

Median age at IO start was 66 (59–74) years, with mUC patients being significantly older than mRCC patients (*p* = 0.034). In 48 mUC patients (71.6%), the primary tumor was localized in the bladder and in 18 patients (26.9%) in the upper urinary tract (UTUC). In mRCC, clear cell morphology was the predominant histology (*n* = 42, 79.2%) and almost half of the patients were defined as poor prognosis per IMDC score. Compared to mRCC patients, mUC patients had more pre-existing malignancies (18.9% vs. 46.3%, *p* = 0.002) in other organ systems. Tumor entities differed significantly in prior systemic therapy (*p* < 0.001), whereas most mUC patients having received one prior therapy (*n* = 37, 55.2%) while most mRCC patients were therapy naive (*n* = 33, 62.3%). Patients received an average of 5 (3–13) IO cycles. The median follow-up was 7 (3–18) months (max. 56 months). Baseline demographics and clinical characteristics are summarized in Table 1.

**Table 1:** Baseline demographics and clinical characteristics of the total cohort and separated by patients with UC and RCC. P values that reach the significance level of 0.05 are written in bold. * p value of Fisher’s exact test

Variables	Total *n* = 120	Urothelial carcinoma *n* = 67	Renal cell carcinoma *n* = 53	*p* Value
**Age at beginning of IO [years]**, median (IQR)	66 (59 − 74)	68 (59 − 77)	63.5 (55.8 − 71)	**0.034**
**Gender**, n (%)				0.143
Male	92 (76.7)	48 (71.6)	44 (83.0)	
Female	28 (23.3)	19 (28.4)	9 (17.0)	
**BMI [kgm**^**2**^**]**, median (IQR)	24.8 (22.9 − 28.0)	25.2 (23.0 − 28.2)	24.5 (22.8 − 27.3)	0.528
**ECOG at beginning of IO**, n (%), *n* = 101				0.606*
0	39 (38.6)	19 (34.5)	20 (43.5)	
1	40 (39.6)	22 (40.0)	18 (39.1)	
2	20 (19.8)	13 (23.6)	7 (15.2)	
3	2 (2.0)	1 (1.8)	1 (2.2)	
≥ 4	0 (0)	0 (0)	0 (0)	
**Tumor entity**, n (%)				-
**Urothelial carcinoma**			-	
Bladder	48 (40.0)	48 (71.6)	-	
UTUC	18 (15.0)	18 (26.9)	-	
CUP	1 (0.8)	1 (1.5)		
**Renal cell carcinoma (RCC)**		-		
Clear cell RCC	42 (35.0)	-	42 (79.2)	
Papillary RCC	8 (6.7)	-	8 (15.1)	
Other histology	3 (2.5)		3 (5.7)	
**Pre-existing malignancies**, n (%)	41 (34.2)	31 (46.3)	10 (18.9)	**0.002**
**Time until onset of metastatic disease [months]**, median (IQR)	5 (0 − 18.5)	6 (0 − 16)	2 (0 − 23.8)	0.457
**IMDC in RCC patients**, n (%), *n* = 45				-
Favorable	8 (17.8)	-	8 (17.8)	
Intermediate	16 (35.6)	-	16 (35.6)	
Poor	21 (46.7)	-	21 (46.7)	
**Number of prior lines of systemic** **therapy**, n (%) **therapy tththerapy**, n (%)				< 0.001*
0	51 (42.5)	18 (26.9)	33 (62.3)	
1	53 (44.2)	37 (55.2)	16 (30.2)	
2	10 (8.3)	9 (13.4)	1 (1.9)	
3	4 (3.3)	3 (4.5)	1 (1.9)	
≥ 4	2 (1.6)	0 (0)	2 (3.8)	
**Number of administered cycles of IO**, median (IQR)	5 (3 − 13)	5 (3 − 13)	6 (3.5 − 11.5)	0.437
**Treatment regime of IO**, n (%)				-
Pembrolizumab	71 (59.2)	52 (77.6)	19 (35.8)	
Nivolumab	28 (23.3)	10 (14.9)	18 (33.9)	
Ipilimumab/Nivolumab	16 (13.3)	-	16 (30.1)	
Atezolizumab	5 (4.2)	5 (7.5)	-	

### Baseline prognostic scores

Median baseline mGPS was 1.5 (0–2), with about one third having a mGPS of 0 (26.7%), about one quarter a mGPS of 1 (23.2%) and half a mGPS of 2 (50.0%). Median SII at baseline was 1224.1 (590.0–2202.7). Median baseline NLR and median baseline NER were 3.9 (2.7–6.8) and 36.1 (16.8–76.0), respectively.

In ROC analysis, SII showed good discrimination between survival and death with an AUC of 0.68. The optimal cut-off value was 979 (Youden Index J: 0.29, sensitivity: 61%, specificity: 67%). ROC analyses for NLR and NER revealed optimal cut-off values of 3.0 (Youden Index J: 0.32, sensitivity: 52%, specificity: 80%, AUC 0.66) and 63.9 (Youden Index J: 0.20, sensitivity: 81%, specificity: 39%, AUC 0.58), respectively.

**Fig. 1:**
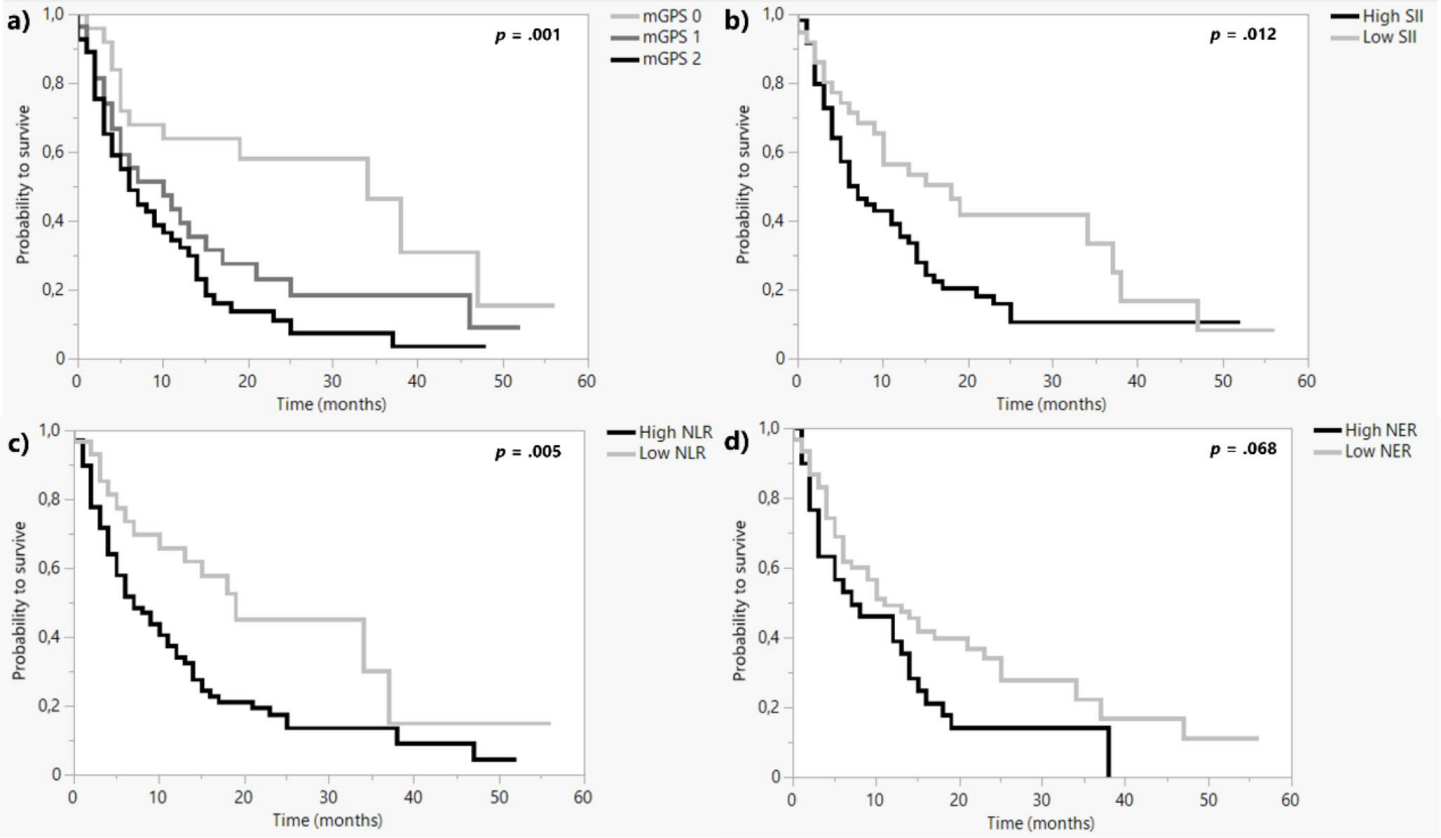
Kaplan-Meier curves for overall survival probability of patients categorized by (a) mGPS, (b) SII, (c) NLR, (d) NER

Figure 1 shows Kaplan-Meier analyses for OS categorized by the four different prognostic scores and revealed that high baseline mGPS, SII and NLR are consistent prognostic factors for poor OS (log rank test *p* < 0.05) with mGPS being the most significant prognostic factor. Patients with low baseline NER survived longer than patients with high NER (11 vs. 7 months, *p* = 0.068).

Figures 2 and 3 in the supplementary section show the Kaplan-Meier analyses for OS in mRCC and mUC patients, respectively, categorized by the four different prognostic scores. High baseline mGPS and NLR significantly predict poor OS in mRCC patients (log-rank test *p* < 0.05), as a high NLR does in mUC patients. High baseline SII predicts a poor OS in both cancer entities with slightly not reaching the significance level (mRCC: log rank test *p* = 0.058; mUC: log rank test *p* = 0.052).

### Predictors of OS

Univariate Cox proportional hazards regression analyses revealed that baseline mGPS, SII, and NLR have significant independent prognostic significance for OS (*p* < 0.05) (Table 2). In multivariate analyses including baseline mGPS, SII, and NLR, only mGPS and NLR were identified as significant factors, with mGPS showing a higher influence. Risk of dying was 4.08 times higher with an mGPS of 2 compared to an mGPS of 0.

**Table 2:** Uni- and multivariate Cox proportional hazards regression analyses of baseline prognostic scores for the prediction of OS. P values that reach the significance level of 0.05 are written in bold. HR Hazard ratio, CI confidence interval

	Univariate			Multivariate			
Variable	HR	95% CI	*p* Value		HR	95% CI	*p* Value
**Baseline mGPS**							
0	Ref				Ref		
1	2.15	1.07 − 4.29	**0.031**		2.45	1.08 − 5.56	**0.032**
2	2.98	1.59 − 5.60	**< 0.001**		4.08	1.90 − 8.75	**< 0.001**
**Baseline SII**							
Low	Ref				Ref		
High	1.88	1.13 − 3.15	**0.016**		0.65	0.31 − 1.35	0.251
**Baseline NLR**							
Low	Ref				Ref		
High	2.24	1.24 − 4.04	**0.010**		2.30	1.06 − 4.99	**0.036**
**Baseline NER**							
Low	Ref						
High	1.56	0.95 − 2.56	0.078				

## Discussion

In recent years, non-invasive immune-inflammatory scores, such as mGPS, SII, NLR and NER, have emerged as easily interpretable and effective tools for predicting tumor prognosis. These scores also gained attention in advanced urological cancers, predicting treatment outcome and patient prognosis, although the conclusions regarding the most convincing parameter are not entirely consistent [5, 12]. Regarding the role of these scores in patients with mUC and mRCC treated with IO, the results of this study showed that high baseline mGPS, SII and NLR were significantly associated with worse OS compared to low baseline values. Furthermore, mGPS and NLR were shown to be independent predictors of OS in our cohort.

Consistent with our results, many studies showed that the NLR is a prognostic marker associated with OS not only in mRCC when treated with IO, but also when treated with anti-vascular endothelial growth factor (VEGF), tyrosine kinase inhibitors (TKIs) and mammalian target of rapamycin (mTOR) inhibition, suggesting an influence on OS regardless of treatment type [13–17]. In a large study analyzing OS and PFS in 422 mRCC patients receiving nivolumab, increasing NLR and SII were associated with disease progression [18].

In bladder cancer, elevated NLR was found to be associated with worse OS, higher tumor stage and lymph node positivity even in the non-metastatic stage [19]. Li et al. observed that the prognostic value of NLR is significant for UC of the bladder, of the upper tract and for mUC [20]. In patients with mUC treated with pembrolizumab, objective response achievement and decline in NLR were independent prognostic factors for improved OS [21]. However, Sahu et al. found high NLR being associated with advanced pathological stage, but could not observe an independent predictive value for recurrence free survival (RFS), PFS and cancer specific survival (CSS) [22].

Compared to the other scores, mGPS is the most extensively studied in urological malignancies. Incorporating albumin and C-reactive protein, mGPS may reflect underlying chronic inflammation, a known risk factor for resistance to IO. However, the results are controversial [23]. In a cohort of 53 mUC patients receiving IO, high mGPS scores were associated with poor OS and PFS [24]. Concerning mRCC, the same group found mGPS comparable to the IMDC risk score in predicting survival outcomes. In their cohort of 156 mRCC patients the median OS was significantly higher with a baseline mGPS score of 0 compared to a score of 2 [25]. Ferro et al. could not confirm a high mGPS to be associated with worse OS and CSS in 1037 patients with UC of the bladder [23]. Only clinico-pathological parameters, such as lymph node positivity and T4 stage were predictors of worse survival. Yet, their data showed a significant association between high mGPS and low RFS. Our results, on the other hand, showed that mGPS is a reliable prognostic score for OS in mRCC and mUC patients receiving IO.

Regarding SII, studies have suggested that higher SII correlates with increased tumor aggressiveness, advanced stage, and poorer OS [26–28]. In a meta-analysis, the prognostic role of SII was analyzed in urological cancers, showing that high SII levels predict poor OS in RCC, UC, prostate cancer and testicular cancer [26]. Interestingly, compared to the NLR, Fornarini et al. stated that SII might be a better predictor of OS and PFS than NLR in advanced UC treated with IO as it incorporates the platelet count [5]. Similar results were observed in non-metastatic patients with UC of the bladder who had undergone radical cystectomy [12]. Preoperative altered SII was significantly associated with higher pathologic stages and worse survival in 2492 patients treated with radical nephroureterectomy for UTUC [28]. However, SII appeared to have relatively limited additive value in clinical use in those patients. Our study revealed similar findings, as high SII was associated with poor OS. However, SII could not be identified as an independent predictor of OS in multivariable analysis compared to the other scores. Similarly, NER had no significant effect on OS in our study and could not be identified as independent predictor of OS. Overall, NER showed the weakest results in our analysis. However, a retrospective review suggested that elevated baseline NER might correlate with worse outcomes in 184 mRCC patients, indicated by decreased OS [29]. Consistently, lower baseline NER was observed to be associated with improved clinical outcomes (PFS, OS, and ORR) in mRCC patients receiving nivolumab/ipilimumab [9]. In line with this, decreased NER three weeks after start of IO was found to be independently associated with significantly longer OS in 125 patients with advanced UC [30]. Regarding this discrepancy, it is important to note that the studies referred to above did not analyze the same prognostic scores as we have done. Thus, the fact that NER performed weakest in our study could be due to the fact that the other scores are even more meaningful.

Overall, given the number of strong studies on the subject, it is reasonable to assume that NLR, mGPS and SII are potent prognostic scores in mUC and mRCC, which has been confirmed in our study.

The findings of our study must be interpreted in the light of its limitations. Firstly, it is a retrospective, single-center study, limiting the generalizability. Additionally, the analyzed cohort is relatively small and includes various tumor entities. In order to give more insights, we nevertheless performed an entity-separated analysis of the prognostic scores, which largely confirmed the strength of the mGPS, SII and NLR. Furthermore, our evaluation focused solely on the prognostic value of the scoring systems at baseline, lacking analyses regarding prediction of PFS and CSS. Nevertheless, we believe that we have provided interesting and clinically relevant insights into easy-to-use scoring systems for patients with metastatic urological malignancies undergoing IO.

## Conclusions

Scoring systems like mGPS, SII, NLR, and NER, utilizing blood biomarkers, offer easy and potentially valuable tools for pretreatment risk assessment in patients with mUC and mRCC. They might be most effective when used in combination with other diagnostic and prognostic tools to provide a comprehensive assessment of patient outcomes. It is crucial to emphasize, that treatment decisions should not solely rely on these scoring systems, as they are still under research. The use of these scores could be a valuable additional tool to engage patients in the discussion about implementing early palliative care programmes. Further research is necessary to standardize their application and fully comprehend their implications across different stages of mUC and mRCC.

## Electronic supplementary material

Below is the link to the electronic supplementary material.

Supplementary Material 1

Supplementary Material 2

## Data Availability

All data generated or analyzed during this study are included in this article. Further enquiries can be directed to the corresponding author.
