# Supplementary figures and images for: Prognostic scores for predicting overall survival in patients with metastatic renal and urothelial cancer undergoing immunotherapy - which one to use?
Source: World J Urol. 2025 Jan 29;43(1):93. doi: 10.1007/s00345-025-05452-4 (PMC11779752; doi:10.1007/s00345-025-05452-4)

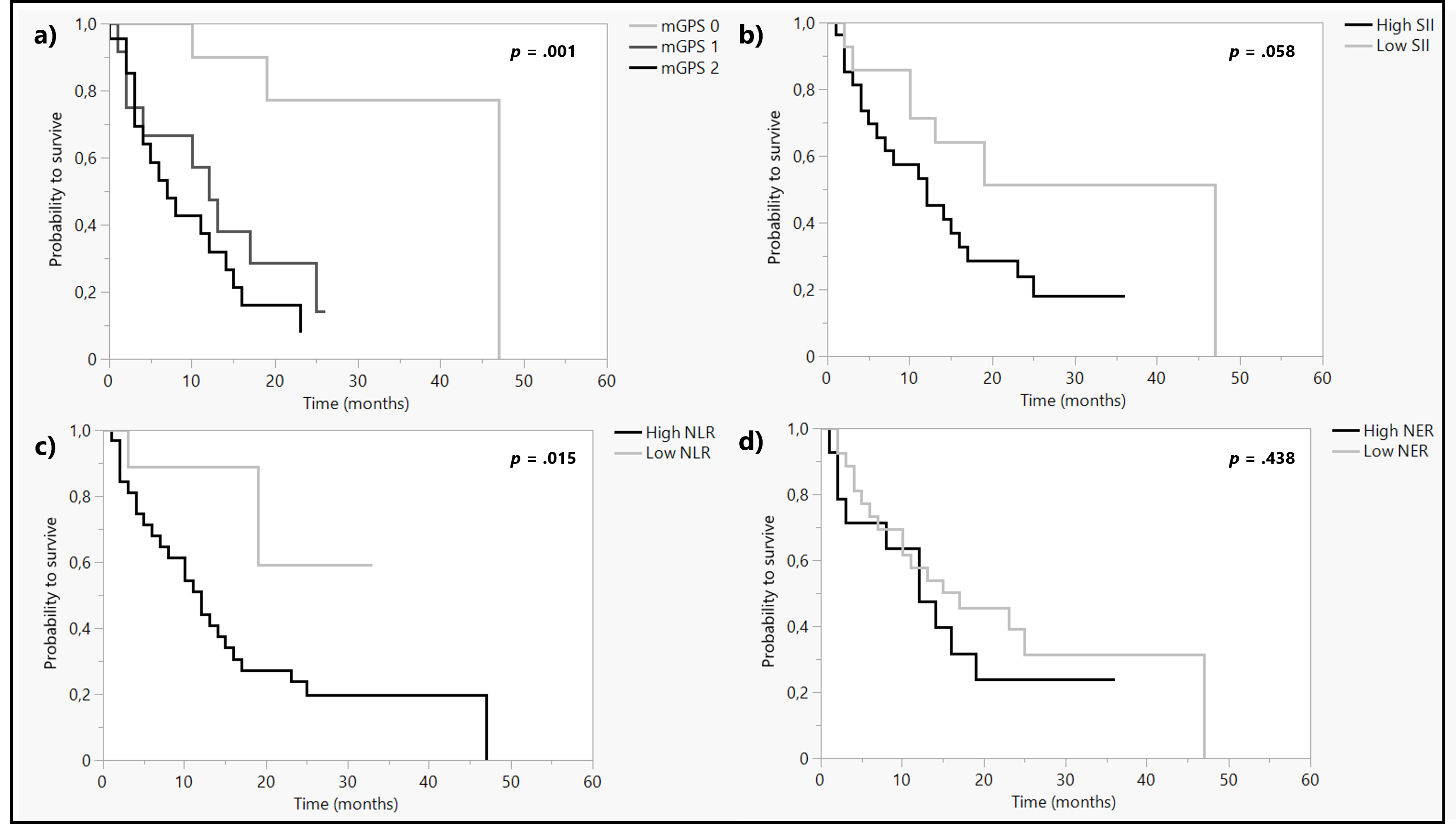

Supplement: Supplementary file 1 — Supplementary Material 1 [file 345_2025_5452_MOESM1_ESM.tif]

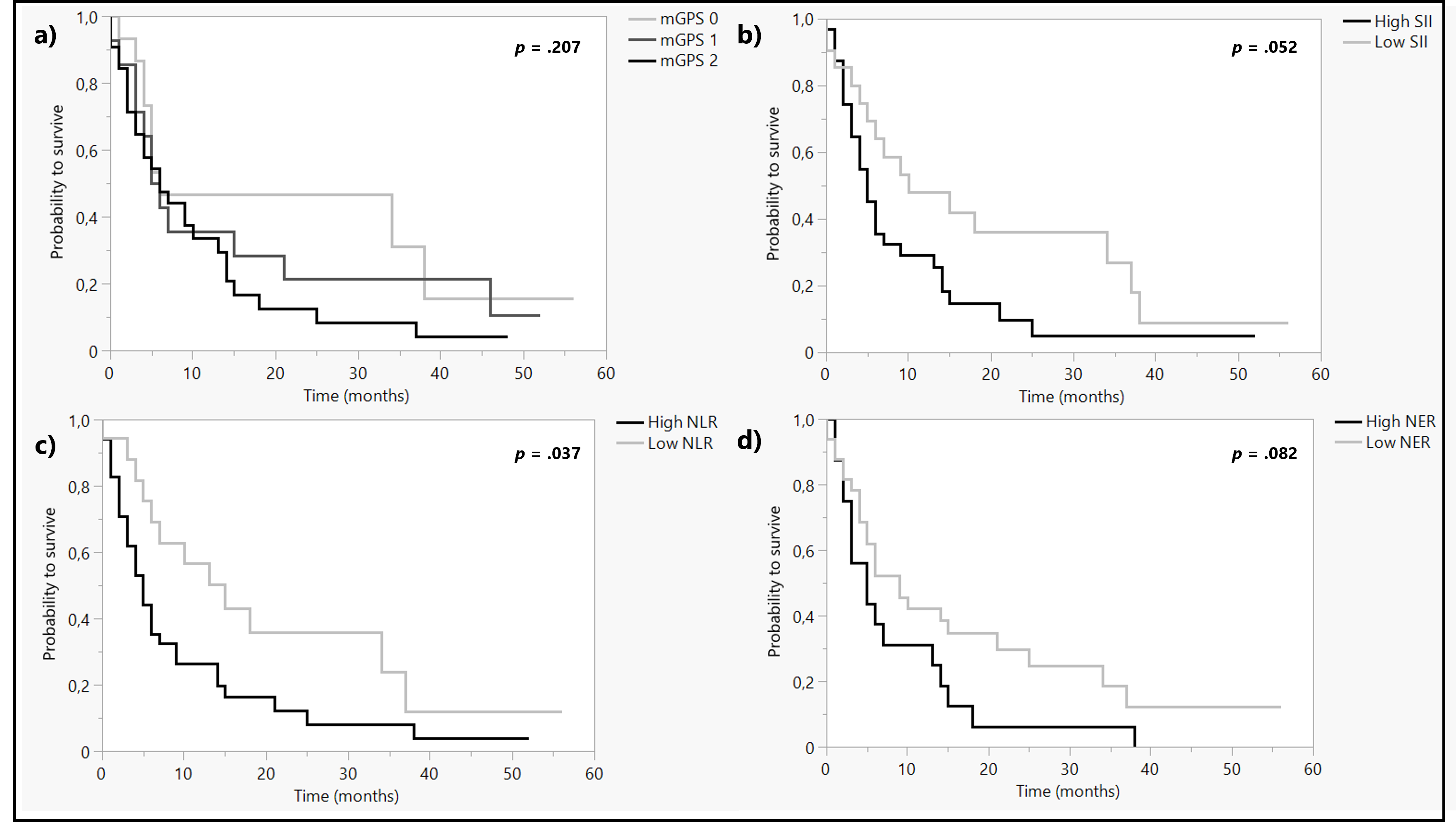

Supplement: Supplementary file 2 — Supplementary Material 2 [file 345_2025_5452_MOESM2_ESM.tif]
